# Elucidating the Solvent‐Dependent Solvation and Structural Stability of Irinotecan: A Molecular Simulation Study

**DOI:** 10.1002/cphc.70359

**Published:** 2026-04-19

**Authors:** Martin M. Bitabo, Sixberth Mlowe, Daniel M. Shadrack, Andrew S. Paluch, Lucas Paul

**Affiliations:** ^1^ Department of Chemistry Faculty of Science Mkwawa University College of Education University of Dar es Salaam Iringa Tanzania; ^2^ Department of Chemistry Dar es Salaam University College of Education Dar es Salaam Tanzania; ^3^ Chemistry Department Faculty of Natural and Applied Sciences St. John's University of Tanzania Dodoma Tanzania; ^4^ Department of Chemical, Paper and Biomedical Engineering Miami University Oxford Ohio USA

**Keywords:** hydrophobic effect, irinotecan, molecular dynamics, solvation free energy, thermodynamics

## Abstract

The clinical application of the chemotherapeutic agent irinotecan is critically hindered by its low and variable solubility. To provide a fundamental understanding of this issue, we employed molecular dynamics simulations and free energy calculations to detail the solvation thermodynamics of irinotecan. Our analysis reveals that irinotecan's solvation preference is governed by a delicate and often competitive balance between two fundamental physical contributions: the Lennard–Jones term (representing cavity formation and dispersion) and favorable solute–solvent electrostatic interactions. We demonstrate that while polar protic solvents (e.g., water) provide the strongest electrostatic stabilization, their high energetic cost for cavity formation severely limits overall solvation favorability. Conversely, polar aprotic solvents (e.g., pyridine and DMSO) optimize this balance by facilitating easier cavity formation while still providing strong electrostatic interactions, resulting in the most favorable solvation profiles. Notably, irinotecan's unexpectedly high relative solubility in cyclohexane compared to water underscores the critical role of solvent reorganization energy in dictating solution‐phase behavior. These molecular‐level findings are rigorously validated by structural analyses (connection matrices and radial distribution functions) and a complementary macroscopic solubility parameter analysis (MOSCED framework). This study offers a robust, integrated, and predictive physicochemical framework for understanding and optimizing the formulation of complex, flexible drug molecules.

## Graphical abstract

The solvation preference of irinotecan is governed by a competition between cavity formation and electrostatic stabilization. Computations reveal that the high cost of breaking water's hydrogen bond network outweighs its electrostatic benefits. In contrast, polar aprotic solvents (e.g., pyridine) offer an optimal thermodynamic balance, providing strong stabilization with a significantly lower cavity penalty.

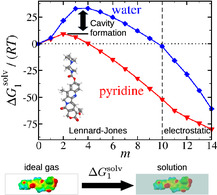

## 1 Introduction

**FIGURE 1:**
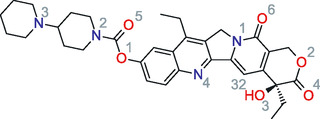
Chemical structure of irinotecan, CAS: 97 682‐44‐5, (S)‐4,11‐diethyl‐3,4,12,14‐tetrahydro‐4‐hydroxy‐3,14‐dioxo1H‐pyrano [3′,4′:6,7]‐indolizino[1,2‐b]quinolin‐9‐yl‐[1,4′bipiperidine]‐1‐carboxylate. Gray atom indices are provided for N 1–4, O 1–6, and H 32 (hydroxyl group H), potential hydrogen bond accepting (N and O) and donating (H) sites, for reference in the discussion of the structural analysis results.

Irinotecan ((S)‐4,11‐diethyl‐3,4,12,14‐tetrahydro‐4‐hydroxy‐3,14‐dioxo1H‐pyrano[3′,4′:6,7]‐indolizino[1,2‐b]quinolin‐9‐yl‐[1,4′bipiperidine]‐1′‐carboxylate), with a molecular formula of C _33_H_38_N_4_O_6_ (see Figure 1), is a semisynthetic camptothecin analog and a crucial chemotherapeutic agent. It functions as a prodrug, enzymatically converted in the body to its active metabolite, SN‐38, which exerts potent anticancer activity by inhibiting the DNA‐topoisomerase I complex [1, 2, 3]. As an effective topoisomerase I inhibitor, irinotecan is used to treat colorectal, lung, and ovarian cancers [4, 5].

Despite its established clinical efficacy, irinotecan faces substantial physicochemical hurdles, including limited and variable solubility and poor absorption [6]. These limitations are rooted in the fundamental thermodynamics of solvation—the complex balance of forces dictating how the solute interacts with its environment. Understanding and predicting irinotecan's solvation behavior in diverse solvent systems is thus a critical step toward rational formulation and delivery optimization. To this end, computational studies have been instrumental, ranging from molecular dynamics (MD) simulations elucidating drug–protein complex stabilization [7] and electronic structure calculations clarifying metabolic kinetics [8, 9], to machine learning approaches predicting pharmacokinetic properties [10, 11].

Despite these advancements, the fundamental physicochemical understanding of irinotecan's structural stability and solvation thermodynamics in various bulk solvent environments remains underexplored. Specifically, the delicate interplay of intermolecular forces and solvent reorganization effects—the two dominant components of the Gibbs free energy of solvation—that govern its solubility is poorly quantified. For instance, while prior work by D’Amelio et al. [12] investigated irinotecan aggregation in dimethyl sulfoxide (DMSO) and noted degradation via lactone ring opening, a comprehensive molecular‐level investigation into how different solvents influence the drug's structural integrity and its precise interaction mechanisms is critical, especially given the known pH dependence of its active lactone conformation [13, 14].

**FIGURE 2:**
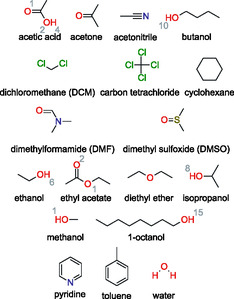
Chemical structures of the organic solvents and water used in the solvation of irinotecan. Gray atom indices are provided for potential hydrogen bond donating and accepting sites for which there is more than one nonsymmetric atom type in the molecule and are provided for reference in the discussion of the structural analysis results.

In this study, we employ a comprehensive computational approach to quantitatively determine the relative solubility and elucidate the underlying molecular and thermodynamic mechanisms of irinotecan solvation. We use extensive MD simulations coupled with free energy calculations to investigate irinotecan in water, 17 pure organic solvents (butanol, ethanol, isopropanol, carbon tetrachloride (CCl_4_), toluene, acetic acid, methanol, acetone, acetonitrile, diethyl ether, ethyl acetate, 1‐octanol, dichloromethane (DCM), pyridine, dimethylformamide (DMF), DMSO, and cyclohexane), and a water‐saturated 1‐octanol binary mixture. The structures of these solvents are depicted in Figure 2. Our approach focuses on two complementary scales:

1. Thermodynamic decomposition: We decompose the solvation free energy into its Lennard–Jones (LJ) (representing cavity formation and dispersion) and Coulombic (electrostatic) components to isolate the balance of forces dictating solvation.
2. Molecular‐level structural analysis: We use connection matrices and radial distribution functions (RDFs) to detail specific solute–solvent interactions and the conformational stability of irinotecan across all environments.

Furthermore, to provide a robust, macroscopic validation of our molecular findings, we correlate our calculated solvation results with a solubility parameter analysis using the MOSCED framework [15, 16, 17, 18, 19]. This comprehensive physical–chemical approach allows us to establish a predictive framework for drug formulation by linking fundamental molecular interactions to macroscopic solubility and permeability metrics.

## 2 Methodology

MD simulations were performed for a single irinotecan molecule in solution, effectively representing the infinite dilution limit where solute–solute interactions are negligible. Previously, Grodowska and Parczewski [20] identified 35 organic solvents and water as being the most common solvents in the pharmaceutical industry, which they broke down into the following 12 classes: alcohols, ketones, halogenated solvents, amide, ethers, sulfur containing, amine, nitriles, esters, aliphatic hydrocarbons, aromatic hydrocarbons, and water. In this study, we investigated 15 pure organic solvents and water, representative of the 12 solvent classes of Grodowska and Parczewski. We additionally considered acetic acid, a common solvent with unique chemical functionality, and 1‐octanol (and water‐saturated 1‐octanol) because of its importance in predicting pharmaceutical behavior. Figure 2 summarizes the chemical structures of the 17 organic solvents and water investigated in this study.

The underlying goal of this work is to understand the solvation mechanism and relative solubility of irinotecan in solvents of varying chemical functionality. This was accomplished through a combination of comprehensive structural analysis and free energy calculations using the multistate Bennett acceptance ratio (MBAR) method [21, 22, 23, 24]. The solvation free energy ($\Delta G_{1}^{\mathrm{solv}}$), where the subscript “1” denotes the solute (irinotecan), is defined as the change in Gibbs free energy when a solute is transferred from a noninteracting ideal gas phase to a solution at the same molar concentration. A negative $\Delta G_{1}^{\mathrm{solv}}$ indicates a preference for the solution phase. Furthermore, the difference in $\Delta G_{1}^{\mathrm{solv}}$ between two solvents directly yields the transfer free energy, which is related to the macroscopically observable relative solubility or the corresponding partition coefficient [25, 26]. Throughout this work, solvation free energies are reported in dimensionless units ($\Delta G_{1}^{\mathrm{solv}}/\left(RT\right)$). This natural thermodynamic scaling directly correlates with the calculation of relative solubility and partition coefficients and aligns seamlessly with the macroscopic solubility formulations (such as MOSCED) employed in our analysis. We also investigated irinotecan's solvation in a water‐saturated 1‐octanol binary mixture to obtain data relevant for determining the 1‐octanol‐water partition coefficient, a crucial metric for drug permeability in biological systems [27].

### 2.1 Molecular Dynamics Simulation

A Class I potential energy functional was used to represent all interactions between the irinotecan and the solvent molecules. Nonbonded intermolecular interactions were accounted for using a combined LJ plus fixed point charge (Coulombic or electrostatic interactions) model [28]:

(1) $$U_{\mathrm{nb}}\left(r_{ij}\right)=4\varepsilon_{ij}\left[{\left(\frac{\sigma_{ij}}{r_{ij}}\right)}^{12}-{\left(\frac{\sigma_{ij}}{r_{ij}}\right)}^{6}\right]+\frac{1}{4\pi \varepsilon_{0}}\frac{q_{i}q_{j}}{r_{ij}}$$

Here, *r*_*ij*_ represents the separation distance between sites *i* and *j*; *ε*_*ij*_ is the well depth of the LJ potential; *σ*_*ij*_ is the distance at which the LJ potential is zero; and *q*_*i*_ and *q*_*j*_ are the partial charges of sites *i* and *j*, respectively.

Irinotecan and the organic solvents were modeled using the General AMBER Force Field version 2 (GAFF2), as implemented in the AMBER 20 simulation suite [29, 30, 31], with AM1‐BCC partial charges [32, 33]. The combination of GAFF and AM1‐BCC partial charges is a well established protocol for predicting solvation thermochemistry in pharmaceutical systems and has demonstrated consistent performance in large‐scale benchmarks [34, 35, 36]. While absolute solvation free energies can be sensitive to the choice of charge model, the current work focuses on identifying the underlying physical mechanisms and relative trends across diverse solvent classes, which are expected to be robust regardless of the specific semi‐empirical charge derivation. Parameters for these molecules were generated using Antechamber and subsequently converted from AMBER to GROMACS format using ParmEd. Initial single molecule 3D structures for irinotecan and all organic solvents were generated with Open Babel 2.3.2 [37, 38]. This initial generation involved a systematic conformational search to identify the lowest energy conformer, followed by geometry optimization (using GAFF and Gasteiger partial charges for this preliminary step with Open Babel). For the MD simulations, water was modeled as rigid using the TIP4P/2005 model for all simulations [39]. This model was selected for its superior ability to reproduce the experimental thermodynamic and structural properties of liquid water compared to other common models. Given the importance of solvent reorganization and cavity formation in our analysis, accurately representing the bulk solvent environment was a priority. This simulation protocol (GAFF2/AM1‐BCC and TIP4P/2005) follows the established and validated workflow used in our previous studies of pharmaceutical solvation and solubility [40, 41, 42], ensuring a consistent and robust thermodynamic description across the diverse solvent environments investigated here.

For all simulations, the number of solvent molecules was chosen to construct a cubic simulation box with an approximate edge length of 4.6 nm at 298.15 K and 1 bar. Initial structures for these systems were generated using Packmol [43]. Subsequently, a 3,000‐step steepest descent minimization was performed to eliminate any poor contacts arising from the packing process. The minimization and all subsequent MD simulations were conducted using GROMACS 2020.2 [44, 45, 46].

The Verlet leap‐frog algorithm was used to integrate the equations of motion for all MD simulations. The systems were equilibrated in a constant number of particles, pressure, and temperature (NPT) ensemble at 298.15 K and 1 bar. This equilibration process proceeded in two distinct stages: initially, a 2 ns run was performed utilizing the Berendsen thermostat and barostat [47, 48, 49]. Subsequently, a longer 12 ns equilibration phase employed the Parrinello–Rahman barostat [50] and the stochastic velocity rescaling thermostat [51, 52, 53].

For irinotecan and the organic solvents, all hydrogen bonds were constrained using the P‐LINCS algorithm [54, 55], while water was modeled as completely rigid using the SETTLE algorithm [56, 57]. LJ interactions were truncated at a 1.4 nm cutoff, with long‐range analytic dispersion corrections applied to the energy and pressure [28, 58, 59]. Lorentz–Berthelot mixing rules were employed for unlike LJ sites [28]. Electrostatic terms were evaluated with the smooth particle‐mesh Ewald (SPME) method [58, 60], with real‐space interactions truncated at 1.4 nm. SPME parameters included a B‐spline order of 4, a Fourier spacing of 0.12 nm, and a relative tolerance between long and short‐range energies of 10^−8^. The equations of motion were integrated with a timestep of 2 fs, and the time constants for the thermostat and barostat were 1 and 4 ps, respectively.

The final structure from this equilibration series served as the initial configuration for our free energy calculations, which will be detailed in the following subsection. For accurate and detailed structural analysis, the equilibration simulations were extended for an additional 100 ns with all 17 pure organic solvents, water, and water‐satured 1‐octanol. The structural analysis was then performed using the final 100 ns trajectory from these extended runs.

### 2.2 Free Energy Calculations

The solvation free energy of irinotecan in each solvent was calculated using the free energy perturbation framework. This approach gradually transforms the solute molecule from a noninteracting, ideal gas state to its fully interacting state in the solvent, and vice versa. This transformation is divided into two distinct, nonoverlapping stages: the decoupling/coupling of the LJ intermolecular interactions and the decoupling/coupling of the intermolecular Coulombic (or electrostatic) interactions. This separation allows for the isolation and quantification of each contribution to the total solvation free energy.

To address the challenges of sampling when the solute is decoupled and weakly coupled to the system, a soft‐core potential was used to couple and decouple the solute–solvent LJ interactions. This potential is important because it allows nearly decoupled molecules to overlap with a finite energy, thereby increasing the phase space overlap between neighboring states. The soft‐core potential had the form [61, 62, 63]:

(2) $$U_{\mathrm{LJ}}^{\mathrm{sc}}\left(r_{ij};m\right)=4\lambda_{m}^{\mathrm{LJ}}\varepsilon_{ij}\{\frac{\sigma_{ij}^{12}}{{\left[\left(1-\lambda_{m}^{\mathrm{LJ}}\right)\alpha_{\mathrm{LJ}}\sigma_{ij}^{6}+r_{ij}^{6}\right]}^{2}}-\frac{\sigma_{ij}^{6}}{\left[\left(1-\lambda_{m}^{\mathrm{LJ}}\right)\alpha_{\mathrm{LJ}}\sigma_{ij}^{6}+r_{ij}^{6}\right]}\}$$

where *α*_LJ_ is a constant with a value of 1/2. The electrostatic term in the intermolecular potential was decoupled linearly as

(3) $$U_{\mathrm{elec}}\left(r_{ij};m\right)=\lambda_{m}^{\mathrm{elec}}\frac{1}{4\pi \varepsilon_{0}}\frac{q_{i}q_{j}}{r_{ij}}$$

For each transformation, a coupling parameter, *λ*, was introduced to control the strength of the nonbonded intermolecular interactions. A total of 15 intermediate states were used for the free energy calculations, where state *m *= 0 corresponds to a noninteracting (ideal gas) state and *m* = 14 is a fully interacting system. The transformation was carried out in two distinct stages. The LJ interactions were gradually coupled in 11 states (from *m *= 0 to *m *= 10), with $\lambda_{m}^{\mathrm{LJ}}$ values ranging from 0.0 to 1.0 in 10 equal increments of 0.1 while $\lambda_{m}^{\mathrm{elec}}=0$. The electrostatic interactions were gradually coupled in 5 states (from *m *= 10 to *m *= 14), with $\lambda_{m}^{\mathrm{elec}}$ values of {0.000.50, 0.71, 0.87, 1.00} following a square root fashion to ensure adequate overlap between states while $\lambda_{m}^{\mathrm{LJ}}=1$.

This separation allows the total solvation free energy ($\Delta G_{1}^{\mathrm{solv}}$) to be expressed as the sum of these two components:

(4) $$\Delta G_{1}^{\mathrm{solv}}=\Delta G_{1}^{\mathrm{solv},\mathrm{LJ}}+\Delta G_{1}^{\mathrm{solv},\mathrm{elec}}$$

where $\Delta G_{1}^{\mathrm{solv},\mathrm{LJ}}$ corresponds to the LJ contribution (the change in free energy from *m *= 0 to *m *= 10) and $\Delta G_{1}^{\mathrm{solv},\mathrm{elec}}$ corresponds to the electrostatic contribution (the change in free energy from *m *= 10 to *m *= 14). The LJ contribution physically represents the free energy change associated with solute cavity formation in solution balanced by solute–solvent dispersion interactions. The electrostatic contribution captures the effect of solute–solvent intermolecular hydrogen bonding and other electrostatic interactions.

For each intermediate state, a 22 ns production run was performed with the initial 2 ns discarded as equilibration. The free energy difference between states was calculated using the MBAR method [21, 22, 23, 24], as implemented in the PyMBAR package [64, 65]. The analysis script implemented an autocorrelation analysis so that only uncorrelated samples were used to determine the free energy and the corresponding uncertainty [66, 67].

The simulation parameters for the free energy calculations were the same as the last step of equilibration, except that the equations of motion were integrated with the GROMACS stochastic dynamics integrator [68]. This change is necessary as a local thermostat is required to correctly control the temperature of the decoupled and weakly coupled solute molecule.

**FIGURE 3:**
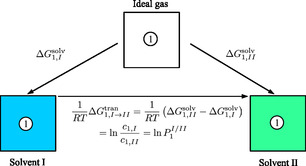
A simple illustration representative of the solvation free energy calculations performed in this work. The solvation free energy of the solute (irinotecan, component 1) is computed in arbitrary solvent *I* and *II*, $\Delta G_{1,I}^{\mathrm{solv}}$ and $\Delta G_{1,II}^{\mathrm{solv}}$, respectively, which is related to the transfer free energy of the solute from solvent *I* to *II*, $\Delta G_{1,I\to II}^{\mathrm{tran}}$. In turn, $\Delta G_{1,I\to II}^{\mathrm{tran}}$ is directly related to to equilibrium concentration (moles/volume or mass/volume) of the solute in solvent *II* and *I*, *c*_1, II_ and *c*_1, I_, respectively, which is directly related to the equilibrium partition coefficient, $P_{1}^{I/II}$, where *R* is the molar gas constant and *T* is the absolute temperature [27, 69].

Free energy calculations were performed for a total of 18 systems (water and 17 pure organic solvents) as well as the water‐saturated 1‐octanol system. From these calculations, one can compute the transfer free energy or relative solubility/partition coefficient of irinotecan between two solvents, as shown in Figure 3.

Sample GROMACS input files are provided in the Supporting Information accompanying the electronic version of this manuscript.

## 3 Results and Discussion

### 3.1 Solvation Free Energy Calculations

The computed values of the total solvation free energy ($\Delta G_{1}^{\mathrm{solv}}$), along with their LJ and electrostatic contributions for irinotecan in water, the 17 pure organic solvents, and water‐saturated 1‐octanol are presented in Table 1, sorted by solvent chemical functionality. The free energy calculations involved systematically coupling/decoupling the solute‐solvent intermolecular interactions in two main phases: the LJ contribution, which physically represents cavity formation and the establishment of nonpolar interactions, and the electrostatic contribution, which corresponds to the establishment of favorable electrostatic interactions, including hydrogen bonding.

**TABLE 1:** A summary of the dimensionless solvation free energy of irinotecan in seventeen pure organic solvents, water, and water‐saturated (sat) 1‐octanol. The solvents are classified by chemical functionality following Ref. [20]. The subscript in the reported solvation free energy corresponds to the statistical uncertainty in the last three decimal places (e.g.,  −33.949_066_ denotes  −33.949 ± 0.066). The solvation free energy is decomposed into the electrostatic (elec, *m* = 10 to 14) and LJ (*m *= 0 to 10) contributions, where the total solvation free energy corresponds to the sum of these two terms. The final column corresponds to the dimensionless transfer free energy of irinotecan from the solvent to water (see Figure 3).

Solvent	Class	CAS	Δ$G_{1}^{\mathrm{solv}}/\left(RT\right)$			Δ$G_{1,I\to \mathrm{water}}^{\mathrm{tran}}/\left(RT\right)$
			elec	LJ	Total	
Methanol	Alcohol	67‐56‐1	−33.949_066_	−38.928_067_	−72.877_094_	11.954_329_
Ethanol	Alcohol	64‐17‐5	−32.636_086_	−45.416_097_	−78.052_130_	17.129_341_
Butanol	Alcohol	71‐36‐3	−30.704_122_	−50.043_134_	−80.747_181_	19.824_363_
1‐Octanol	Alcohol	111‐87‐5	−21.828_082_	−52.921_276_	−74.749_288_	13.826_427_
1‐Octanol (sat)	Alcohol		−18.170_040_	−53.511_304_	−71.681_307_	10.758_440_
Isopropanol	Alcohol	67‐63‐0	−30.692_096_	−48.178_128_	−78.870_160_	17.947_353_
Acetone	Ketone	67‐64‐1	−26.992_045_	−47.425_083_	−74.418_094_	13.495_329_
Dichloromethane	Halogenated	75‐09‐2	−24.021_035_	−52.869_074_	−76.891_082_	15.968_325_
Carbon tetrachloride	Halogenated	56‐23‐5	−19.441_041_	−58.312_099_	−77.754_107_	16.831_333_
Dimethylformamide	Amide	68‐12‐2	−31.150_089_	−47.315_155_	−78.466_179_	17.543_362_
Diethyl ether	Ether	60‐29‐7	−20.457_038_	−51.711_073_	−72.167_082_	11.244_325_
Dimethyl sulfoxide	Sulfur	67‐68‐5	−34.080_098_	−43.364_182_	−77.444_206_	16.521_376_
Pyridine	Amine	110‐86‐1	−28.034_059_	−51.996_123_	−80.031_137_	19.108_344_
Acetonitrile	Nitrile	75‐058	−26.554_037_	−48.548_081_	−75.102_089_	14.179_327_
Ethyl acetate	Esters	141‐78‐6	−27.041_054_	−49.823_117_	−76.864_129_	15.941_340_
Cyclohexane	Aliphatic	110‐82‐7	−15.544_063_	−56.228_147_	−71.772_160_	10.849_353_
Toluene	Aromatic	108‐88‐3	−22.947_060_	−55.059_103_	−78.005_119_	17.082_337_
Acetic acid	Carboxylic acid	64‐19‐7	−37.590_130_	−43.797_180_	−81.387_222_	20.464_385_
Water	Water	7732‐18‐5	−58.449_102_	−2.474_298_	−60.923_315_	

Overall, the total solvation free energy reflects a delicate balance between these two competing factors. Our results indicate that the overall $\Delta G_{1}^{\mathrm{solv}}$ is most favorable for pyridine (–80.028 *RT*), followed closely by DMSO (–74.373 *RT*). In contrast, water's overall solvation is significantly less favorable (–60.923 *RT*), despite its highly polar nature.

The LJ contribution is a measure of the free energy cost of creating a cavity to accommodate the solute, plus weak solute–solvent dispersion interactions. Consistent with its physical interpretation, we observe that this contribution is least favorable (least negative) for irinotecan in water (–2.475 *RT*). This large unfavorable penalty indicates the significant difficulty and energetic cost of forming a cavity in water, which requires breaking its strong, extensive hydrogen bonding network [70]. Conversely, cyclohexane, a nonpolar solvent, demonstrates the most favorable (most negative) LJ contribution (–27.910 *RT*). This is largely because its weaker intermolecular interactions facilitate easy disruption of its molecular arrangement and reduce the free energy change needed to produce cavities. The polar aprotic solvents, such as pyridine and DMSO, exhibit an intermediate LJ contribution (–16.920 *RT* and –17.610 *RT*, respectively), which is more favorable than that of water, indicating easier cavity formation due to the absence of solvent self‐association in our polar aprotic solvents.

The electrostatic contribution quantifies the strength of favorable hydrogen bonding and other electrostatic interactions. We found this contribution to be most favorable in highly polar protic solvents such as water (–58.448 *RT*) and methanol (–56.226 *RT*), due to significant hydrogen bonding interactions. Polar aprotic solvents, including DMSO (–56.763 *RT*) and pyridine (–51.621 *RT*), also show highly favorable electrostatic contributions comparable to the polar protic solvents. This result is interesting as from Figure 1, the number of potential hydrogen bond accepting sites in irinotecan far outnumber the *single* potential hydrogen bonding site (hydroxyl hydrogen). This would suggest that the irinotecan hydroxyl groups acts as a strong hydrogen bond donor and is a major interaction site in the system. On the other hand, the nonpolar and weakly polar solvents such as cyclohexane (–15.544 *RT*) and DCM (–24.022 *RT*) have less favorable electrostatic contributions, as their interactions with irinotecan are primarily driven by dispersion forces.

The overall solvation free energy directly reflects the combined effect of these two competing factors. While water exhibits the most favorable electrostatic contribution, its significantly unfavorable LJ contribution severely limits its overall solvation favorability. Conversely, polar aprotic solvents such as pyridine and DMSO achieve the most favorable overall solvation because they strike an optimal balance: they offer a moderately favorable LJ contribution (easier cavity formation than water and the polar protic solvents) while simultaneously providing a highly favorable electrostatic contribution. This highlights that while strong solute–solvent electrostatic interactions are crucial, their favorability is always counterbalanced by the free energy cost associated with overcoming solvent–solvent interactions and forming a cavity to accommodate the solute. This also explains in part why irinotecan's solubility is greater in cyclohexane than in water, as the ease of cavity formation in cyclohexane outweighs water's much higher electrostatic contribution.

**FIGURE 4:**
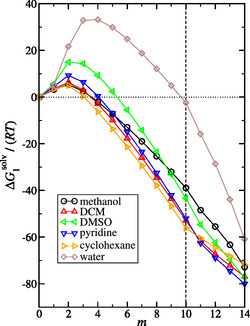
Plot of the calculated dimensionless solvation free energy of irinotecan versus *λ*‐state (*m*) for the solvents methanol, DCM, DMSO, pyridine, cyclohexane, and water, as labeled. The vertical dashed line corresponds to *m* = 10, and the horizontal dotted line corresponds to $\Delta G_{1}^{\mathrm{solv}}/(RT)=0$ and are drawn as a reference. Recall the LJ coupling parameter ($\lambda_{m}^{\mathrm{LJ}}$) was increased from 0 to 1 from *m* = 0 to *m* = 10, and the electrostatic (elec) coupling parameter ($\lambda_{m}^{\mathrm{elec}}$) was increased from 0 to 1 from *m* = 10 to *m* = 14.

In Figure 4, we plot the computed solvation free energy as a function of *λ*‐state *m* for the representative systems discussed in this section. We find that for all systems the solvation free energy initially increases and goes through a maximum. A positive solvation free energy indicates that the noninteracting ideal gas state is preferred relative to solution, with the larger the value the more unfavorable. We attribute this to cavity formation. We find that this first peak in water far exceeds the other solvents, emphasizing the challenge of cavity formation in water. After the peak, the solvation free energy decreases first due to favorable dispersion and subsequently electrostatic (i.e., association) interactions. While water exhibit the most favorable electrostatic contribution to the solvation free energy, it is not able to overcome the highly unfavorable cavity formation process.

### 3.2 Solubility Parameters

As confirmation of the observed mechanism, we will make use of the solubility parameter method Modified Separation of Cohesive Energy Density (MOSCED) [15, 16, 17, 18, 19]. Due to their capacity to predict phase equilibrium and elucidate intermolecular interactions, solubility parameter‐based methods have been a staple in early‐stage process conceptualization and design [71, 72, 73]. Two of the most common solubility parameter methods are the Hildebrand and Hansen Solubility Parameter methods [72, 73]. Hansen differs from Hildebrand in that it separates the solubility parameter into dispersion, polar, and hydrogen bonding interactions. MOSCED differs from Hansen in that it further separates the hydrogen bonding term into independent contributions due to hydrogen bond donating and accepting ability, resulting in an improved description of association in solution [17]; a similar improvement has been suggested for the Hansen Solubility Parameter method [74].

**FIGURE 5:**
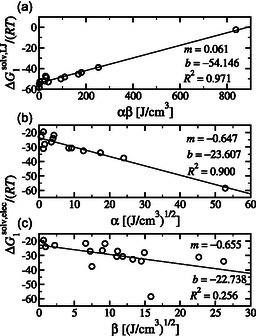
Plots of the calculated contribution to $\Delta G^{\mathrm{solv}}$ versus the hydrogen bonding MOSCED parameters of the solvents, where *α* and *β* correspond to hydrogen bond donating and accepting strength, respectively. In (a), we plot the LJ contribution versus the self‐association strength of the solvent. In (b) and (c), we plot the electrostatic (elec or Coulombic) contribution versus the hydrogen bond accepting and donating strength of the solvent, respectively. The straight line corresponds to the line of best fit, where *m* and *b* correspond to the slope and intercept, respectively.

Essentially, within MOSCED, a single molecular descriptor is assigned to a molecule to quantify its overall dispersion (*λ*), polar (*τ*), and hydrogen bond donating (*α*) and accepting (*β*) interactions. In Figure 5, we plot the contributions of $\Delta G^{\mathrm{solv}}$ of irinotecan versus the solvent hydrogen bonding MOSCED parameters. In the top pane (a), we plot the LJ contribution versus the MOSCED self‐association strength (*α *× *β*) of the solvent. We find that as the self‐association strength of the solvent increases, the LJ contribution to $\Delta G^{\mathrm{solv}}$ increases, or becomes less favorable. As discussed earlier, the LJ contribution accounts for the solute transitioning from a noninteracting ideal gas state to a solute with LJ interactions but no electrostatic interactions. During this phase, a cavity is created in the solvent due to the displacement of solvent molecules by the solute, resulting in the separation of solvent–solvent interactions. It stands that the stronger the self‐association strength of the solvent, the greater the challenge to create the cavity, leading to a larger LJ contribution.

In the middle (b) and bottom (c) panes, we plot the electrostatic (or Coulombic) contribution versus the solvent MOSCED hydrogen bond donating (*α*) and accepting (*β*) strength. As discussed earlier, the electrostatic contribution accounts for the transition from full LJ without electrostatic interactions to full LJ with electrostatic interactions. During this phase, we account for the association between the solute and solvent. We find that as the hydrogen bond donating and accepting strength of the solvent increases, the electrostatic contribution of $\Delta G^{\mathrm{solv}}$ decreases or becomes more favorable. We observe the same trend for both the hydrogen bond donating and accepting strengths of the solvent, supporting our observation that irinotecan acts as both a hydrogen bond donor and acceptor in its interactions with the solvent. Noticeably, the correlation is significantly stronger with the solvent hydrogen bond donating strength (*α*) as compared to the solvent hydrogen bond accepting strength (*β*). As shown in the chemical structure of irinotecan (see Figure 1), it has 10 sites capable of accepting hydrogen bonds, and only 1 site capable of donating a hydrogen bond. We hypothesize that the difference may result from the availability of complementary hydrogen bonding interactions. This is further interesting as we previously observed that the electrostatic contribution was most favorable in highly polar protic solvents such as water (–58.448 *RT*) and methanol (–56.226 *RT*), which are capable of both donating and accepting hydrogen bonds, as compared to the slightly lower values in polar aprotic solvents, including DMSO (–56.763 *RT*) and pyridine (–51.621 *RT*), which are capable of only accepting hydrogen bonds. Despite irinotecan having just a single hydrogen bond‐donating site, it has a major impact on the observed solvation.

Note that within Figure 5, cases where (a) *αβ* = 0, (b) *α* = 0, and (c) *β* = 0 were excluded. We additionally excluded diethyl ether and water‐saturated 1‐octanol for which MOSCED parameters do not exist.

**FIGURE 6:**
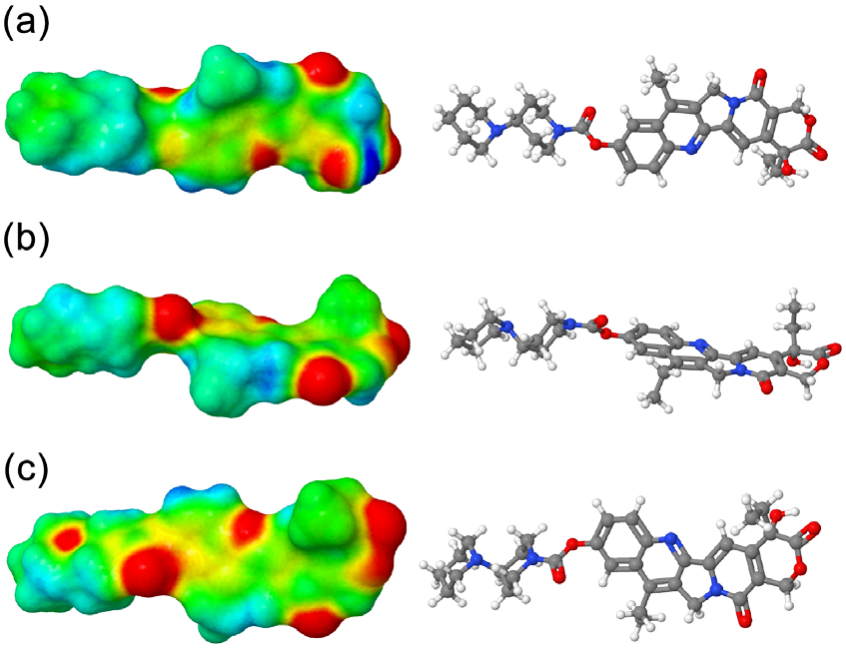
A surface illustrating the electrostatic potential distribution of irinotecan. Low to high potential goes from red to blue. Regions of low potential (red) correspond to hydrogen bond accepting sites, and regions of high potential (deep blue) correspond to hydrogen‐bond donating sites. The labels (a), (b), and (c) indicate unique view angles.

Next, Figure 6 contains a surface illustrating the partial atomic charge distribution of irinotecan. While we do not have MOSCED parameters for irinotecan, Figure 6 illustrates the presence of both hydrogen bond accepting (red) and donating (deep blue) sites, albeit with a much smaller number of donating sites. This reiterates our observation that irinotecan acts as both a hydrogen bond donor and acceptor in its interactions with the solvent, with many more hydrogen bond accepting sites available.

### 3.3 Transfer Free Energy

The dimensionless transfer free energy, $\Delta G_{1,I\to \mathrm{water}}^{\mathrm{tran}}$, of irinotecan (1) from the studied solvents (*I*) to water is tabulated in Table 1 and is directly related to the log relative solubility of irinotecan in the studied solvent relative to water (or log partition coefficient, see Figure 3). The transfer free energy to water provides valuable insights into its solubility, hydrophobic interactions, and potential pharmacokinetic implications. A value of $\Delta G_{1,I\to \mathrm{water}}^{\mathrm{tran}}>0$ indicates a preference of irinotecan for the studied solvent relative to water, where the greater the value, the greater the preference. We additionally emphasize that the solubility relative to water is exponentially related to this term.

We find that in all cases, $\Delta G_{1,I\to \mathrm{water}}^{\mathrm{tran}}>0$. The observed values range from 10.758 *RT* in water‐saturated 1‐octanol to 20.464 *RT* in acetic acid, demonstrating a broad variation that reflects the diverse solute–solvent interactions. The large average positive value can be attributed to the strong water–water self‐association interactions and the associated thermodynamic challenge of cavity formation in water. The LJ contribution to the total solvation free energy in water is –2.474 *RT*, approximately 36 *RT* greater (less negative) than the next closest value (–38.928 *RT* for methanol). Despite water's most favorable electrostatic contribution, the strong irinotecan–water interactions are unable to overcome the penalty imposed by strong water–water self‐interactions.

Interestingly, $\Delta G_{1,I\to \mathrm{water}}^{\mathrm{tran}}$ increases by approximately 3 *RT* in going from water‐saturated 1‐octanol to neat (pure) 1‐octanol. Within neat 1‐octanol, the system exhibits microscale heterogeneities composed of domains formed by the polar head groups (hydroxyl groups) and the nonpolar tails. With the addition of water, the polar domain size increases, which is reflected here as less favorable solvation (lower preference compared to pure octanol). Interestingly, here the difference is found in the electrostatic contribution, further highlighting the competition between irinotecan–solvent association and solvent–solvent hydroxyl group association (OH from water or octanol). The case of 1‐octanol is unique in that the initial cavity (within the LJ contribution) may form in the nonpolar domain, offering a distinct solvation environment [27, 75, 76, 77].

Considering the homologous series of linear alcohols, we find that in going from methanol to 1‐octanol, the LJ contribution to the total solvation free energy decreases (becomes more negative), suggesting easier cavity formation and greater dispersion interactions. At the same time, the electrostatic contribution increases (becomes less negative). We find that $\Delta G_{1,I\to \mathrm{water}}^{\mathrm{tran}}$ increases from methanol to butanol, with the greatest increase going from methanol to ethanol, but then the value decreases in going from butanol to 1‐octanol. This further emphasizes the balance of interactions driving solvation. We seek strong irinotecan–solvent (association/hydrogen bonding) interactions, but this must be balanced against the strength of the solvent–solvent self‐interactions.

### 3.4 Structural Analysis

To gain a comprehensive overview of the specific solute–solvent and solvent–solvent hydrogen bonding interactions, we performed a connection matrix (CMat) analysis [78, 79] followed by an analysis of the RDFs.

#### 3.4.1 Connection Matrix Analysis

Generally, the CMat consists of two components: the left panel displays the interaction connectivity between potential proton donors and acceptors, while the right panel serves as a heat map quantifying the interaction strength. This strength is defined by the intensity of the first peak of the local density (i.e., the unnormalized RDF); higher intensity at shorter distances indicates a stronger interaction. A matrix element marked with a black cross indicates no interactions within a cutoff distance of 350 pm. In the matrices presented below, potential hydrogen bond accepting sites of irinotecan and the solvent are plotted on the rows, while potential hydrogen bond donating sites are plotted on the columns. We refer to the atom indices defined in Figures 1 and 2.

**FIGURE 7:**
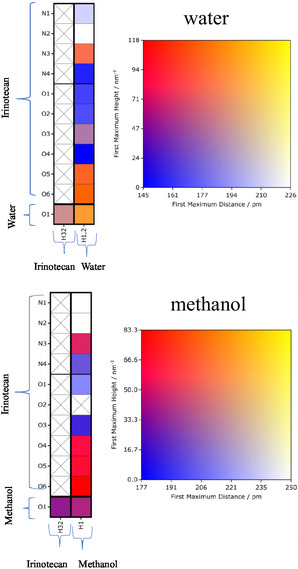
The CMat analysis of irinotecan in water and methanol, as labeled. The intensity corresponds to the peak height of the local density (unnormalized RDF) within a 350 pm cutoff. See Figures 1 and 2 for atom indices.

First, in Figure 7, we consider the solvents water and methanol. For water, the two symmetric hydrogens are grouped as H1‐2. We observe strong interactions between the water hydrogens and two of irinotecan's carbonyl groups (O5 and O6), followed closely by N3. Interactions between the water hydrogens and irinotecan's hydroxyl oxygen (O3) are present but are noticeably weaker than those with O5, O6, and N3. Crucially, we observe very favorable water–water (H–O) interactions. This structural evidence supports the free energy results, which suggested a competitive balance between irinotecan–solvent and solvent–solvent interactions. Interactions between the single potential hydrogen bond donating site of irinotecan (H32, hydroxyl H) and water (O) are also present but appear weaker than the interactions where water acts as the donor.

The results for methanol are similar, with two notable differences. First, the carbonyl oxygen O4 of irinotecan also interacts favorably with the methanol hydrogen. Second, the intensity of the methanol–methanol (H–O) interaction is significantly lower than that of water–water. This validates the thermodynamic argument that cavity formation in water requires overcoming much stronger solvent–solvent associations compared to methanol, explaining water's highly unfavorable LJ contribution to the solvation free energy.

**FIGURE 8:**
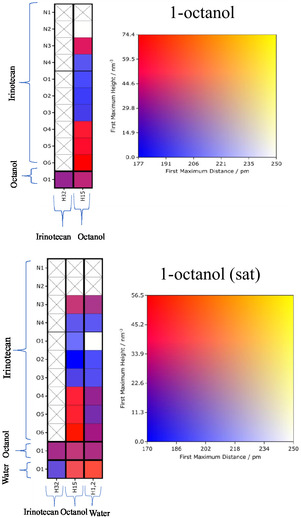
The CMat analysis of irinotecan in 1‐octanol and water‐saturated 1‐octanol, as labeled. See Figures 1 and 2 for atom indices.

Next, in Figure 8, we consider 1‐octanol and water‐saturated 1‐octanol. The interaction pattern in pure 1‐octanol closely resembles that of methanol. In the water‐saturated 1‐octanol mixture, the relative favorability of irinotecan–octanol and octanol–octanol interactions remains similar to the pure solvent. However, we identify a critical competitive mechanism: favorable water–octanol (O–H) interactions emerge, particularly where water acts as the hydrogen bond acceptor (O) and either 1‐octanol or water acts as the donor. This competition—where water and 1‐octanol essentially solvate each other rather than the solute—provides a structural explanation for the less favorable electrostatic contribution observed in the binary mixture compared to pure 1‐octanol.

**FIGURE 9:**
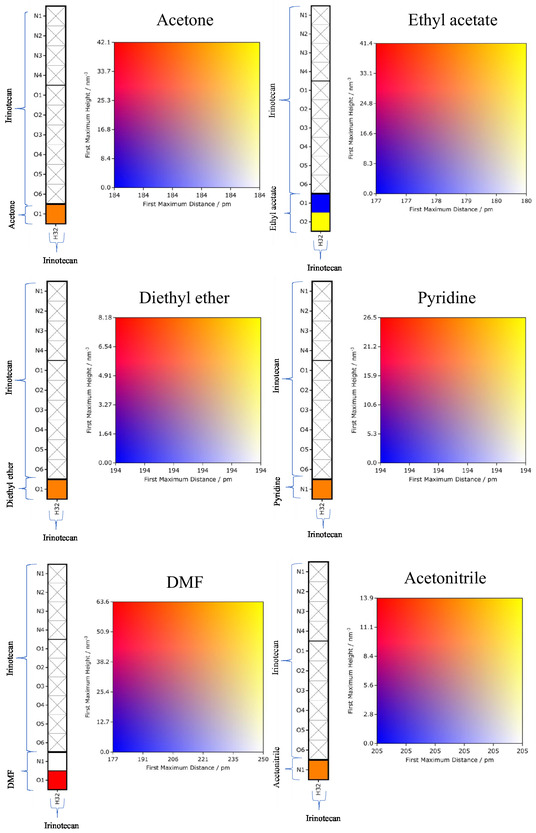
The CMat analysis of irinotecan in select polar aprotic solvents. The rows correspond to the hydrogen bond accepting sites of the solvent, and the columns correspond to the single hydrogen bond donating site of irinotecan (H32).

Finally, in Figure 9, we consider irinotecan in polar aprotic solvents, which are capable of accepting but not donating hydrogen bonds. Since irinotecan contains only a single potential hydrogen bond donating site (the hydroxyl hydrogen, H32), all hydrogen bonding interactions in these systems occur between H32 and the solvent's acceptor site. Consistent with the electrostatic free energy contributions, DMF exhibits the highest interaction intensity, while diethyl ether shows the lowest.

#### 3.4.2 Radial Distribution Functions

**FIGURE 10:**
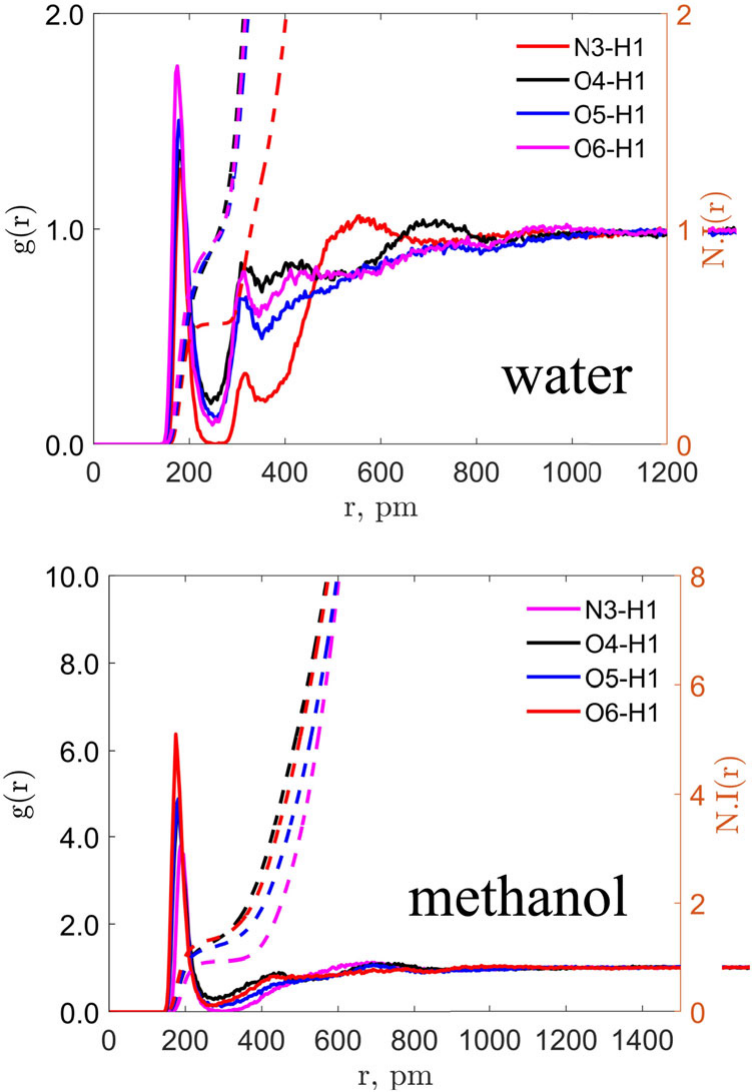
RDF and number integral analysis for irinotecan in water and methanol, as labeled. See Figures 1 and 2 for atom indices. The first site corresponds to irinotecan, and the second is H1 of water or methanol.

We next quantify these interactions using the RDF, *g*(*r*), and its corresponding number integral, N.I.(r). While the CMat heat map visualizes unnormalized local density, the RDF represents the local density relative to the bulk density, providing a normalized probability of finding an atom at a given distance *r*. In Figure 10, we examine water and methanol, focusing on the strongest interactions identified by the CMat analysis: irinotecan as a hydrogen bond acceptor (N3, O4, O5, O6) and the solvent as the donor. For the specific case of water, we calculate the RDF using only one of the equivalent hydrogen atoms (H1), rather than aggregating both as in the CMat analysis. This facilitates a direct, site‐to‐site comparison with the single hydroxyl hydrogen of methanol. In both solvents, the interaction with the carbonyl oxygen O6 is the most intense. Notably, the peak intensity for O6 in methanol is over three times greater than in water. This difference arises partly from normalization (bulk density of water is higher) but also highlights the specificity of the interaction in methanol.

**FIGURE 11:**
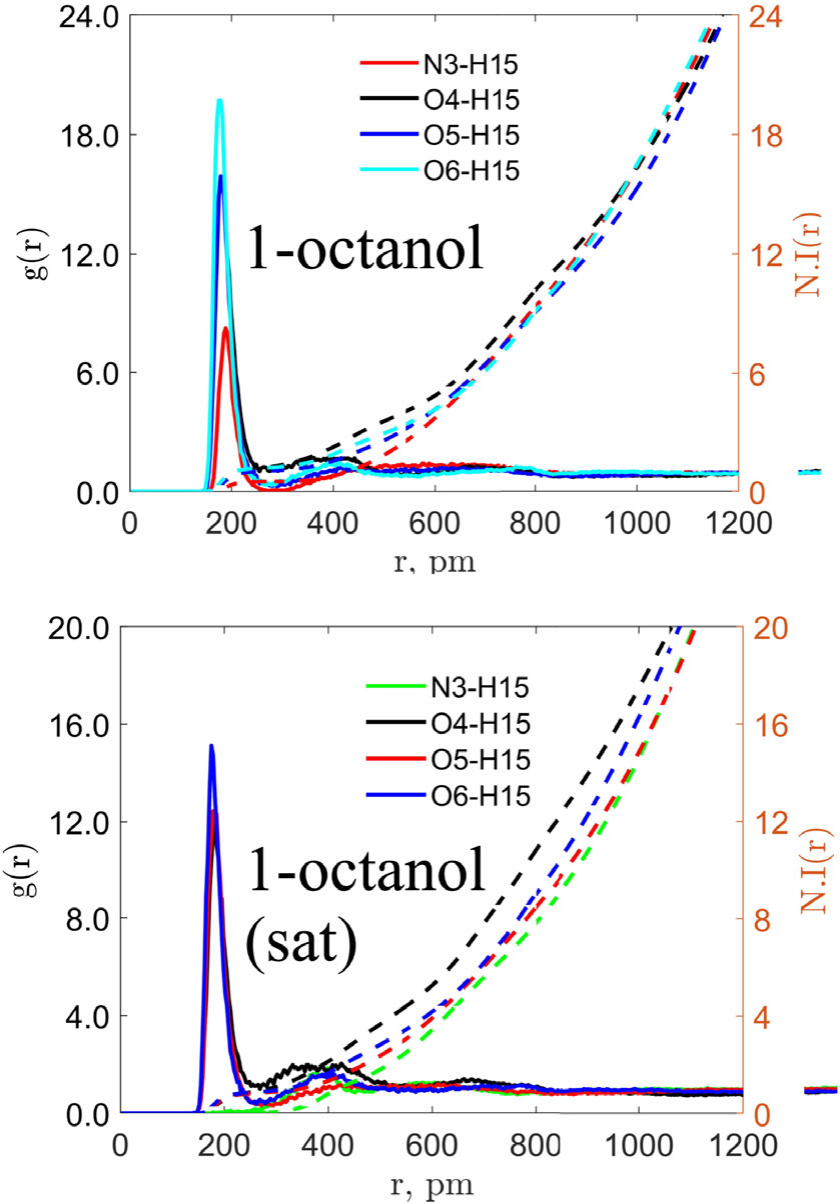
RDF and number integral analysis for irinotecan in 1‐octanol and water‐saturated 1‐octanol, as labeled. See Figures 1 and 2 for atom indices. The first site corresponds to irinotecan, and the second is H1 of 1‐octanol.

In Figure 11, we consider 1‐octanol and the water‐saturated mixture, again analyzing irinotecan acceptors with the solvent donor (H15). Consistent with the water and methanol results, O6 remains the dominant interaction site. We observe a trend in peak intensity for O6: 1‐octanol > methanol > water. This trend inversely correlates with the bulk number density of solvent hydroxyl groups (1‐octanol < methanol < water), suggesting that in solvents with lower hydroxyl density, the specific solvation of irinotecan's carbonyls becomes structurally more pronounced relative to the bulk. Upon saturation with water, the O6 peak intensity in 1‐octanol decreases slightly, but a more significant drop‐off is observed for N3, further evidencing the competitive solvation effects discussed previously.

**FIGURE 12:**
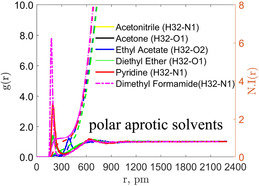
RDF and number integral analysis for irinotecan in select polar aprotic solvents, as labeled. See Figures 1 and 2 for atom indices. The first site corresponds to the hydroxyl hydrogen of irinotecan (H32), and the second is the hydrogen bond accepting site of the solvent.

Finally, Figure 12 presents the RDFs for the polar aprotic solvents, focusing on the interaction between irinotecan's donor (H32) and the solvent's acceptor. In agreement with the CMat analysis, we find significant variation in peak intensities. DMF displays the highest peak intensity, correlating directly with its highly favorable electrostatic contribution to the solvation free energy. This confirms that for aprotic solvents, the strength of this single hydrogen bond (H32 … Solvent) is a critical determinant of thermodynamic stability.

## 4 Conclusion

This study investigated the solvation of the pharmaceutical compound irinotecan in a diverse set of organic solvents, water, and a water‐saturated 1‐octanol binary mixture using MD simulations and free energy calculations. Our primary objective was to elucidate the underlying molecular mechanisms and thermodynamic driving forces governing irinotecan's solvation preference.

The total solvation free energy ($\Delta G_{1}^{\mathrm{solv}}$) calculations revealed that irinotecan exhibits the most favorable solvation in pyridine and DMSO. This stands in contrast to water, which, despite its high polarity, showed a significantly less favorable overall solvation free energy. This observation is attributed to the delicate balance between two competing factors: the LJ contribution, representing the free energy cost of cavity formation and dispersion interactions, and the electrostatic contribution, representing favorable specific associations. While water provided the most favorable electrostatic stabilization, the high thermodynamic penalty of forming a cavity within its extensive hydrogen bonding network rendered its overall solvation less favorable.

Our detailed structural analysis, employing CMat and RDF techniques, provided a microscopic explanation for these thermodynamic findings. The results demonstrated that polar aprotic solvents, such as pyridine and DMSO, are particularly effective at solvating irinotecan. They strike an optimal balance by facilitating strong electrostatic stabilization through specific hydrogen bonding interactions while simultaneously requiring significantly less free energy for cavity formation compared to water. This explains their superior ability to solvate irinotecan relative to both highly protic solvents (like water) and nonpolar solvents (like cyclohexane). Furthermore, the analysis of the water‐saturated 1‐octanol system highlighted the competitive solvation effects between water and octanol, providing valuable insight into the partitioning behavior of irinotecan—a property critical for predicting drug absorption and biodistribution.

These findings are further validated by a complementary solubility parameter analysis using the MOSCED framework, which provided macroscopic confirmation of the observed molecular trends. The methodology presented in this work is broadly applicable and can be utilized to screen for optimal solvents in drug formulation, crystallization, and purification processes. By quantifying the free energy contributions and correlating them with atomic‐level structural details, we have established a robust framework for understanding and predicting the solvation behavior of complex pharmaceutical molecules in diverse solvent environments [80].

## Supporting Information

Additional supporting information can be found online in the Supporting Information section. We provide sample input files to reproduce the results of the present study as a zip in the supporting information accompanying the electronic version of this article. Additional information and details may be made available by contacting the corresponding author.

## Author Contributions

**Martin M. Bitabo**: conceptualization, methodology, software, validation, formal analysis, investigation, resources, data curation, writing – original draft, writing – review & editing, visualization, project administration, funding acquisition. **Sixberth Mlowe**: conceptualization, methodology, resources, writing – review & editing, supervision, project administration, funding acquisition. **Daniel M. Shadrack**: conceptualization, methodology, software, validation, formal analysis, investigation, resources, data curation, writing – original draft, writing – review & editing, visualization, supervision, project administration, funding acquisition. **Andrew S. Paluch**: conceptualization, methodology, software, validation, formal analysis, investigation, resources, data curation, writing – original draft, writing – review & editing, visualization, supervision, project administration, funding acquisition. **Lucas Paul**: conceptualization, methodology, software, validation, formal analysis, investigation, resources, data curation, writing – original draft, writing – review & editing, visualization, supervision, project administration, funding acquisition.

## Conflicts of Interest

The authors declare no conflicts of interest.

## Supporting information

Supplementary Material

## Data Availability

The data that supports the findings of this study are contained in this article.
